# Tumour risk associated with use of cellular telephones or cordless desktop telephones

**DOI:** 10.1186/1477-7819-4-74

**Published:** 2006-10-11

**Authors:** Lennart Hardell, Kjell Hansson Mild, Michael Carlberg, Fredrik Söderqvist

**Affiliations:** 1 Department of Oncology, University Hospital, SE-701 85 Örebro and Department of Natural Sciences, Örebro University, SE-701 82 Örebro, Sweden; 2 National Institute for Working Life, SE-907 13 Umeå and Department of Natural Sciences, Örebro University, SE-701 82 Örebro, Sweden; 3 Department of Oncology, University Hospital, SE-701 85 Örebro, Sweden; 4 Department of Oncology, University Hospital and Institute of Clinical Medicine, Örebro University, SE-701 85 Örebro, Sweden

## Abstract

**Background:**

The use of cellular and cordless telephones has increased dramatically during the last decade. There is concern of health problems such as malignant diseases due to microwave exposure during the use of these devices. The brain is the main target organ.

**Methods:**

Since the second part of the 1990's we have performed six case-control studies on this topic encompassing use of both cellular and cordless phones as well as other exposures. Three of the studies concerned brain tumours, one salivary gland tumours, one non-Hodgkin lymphoma (NHL) and one testicular cancer. Exposure was assessed by self-administered questionnaires.

**Results:**

Regarding acoustic neuroma analogue cellular phones yielded odds ratio (OR) = 2.9, 95 % confidence interval (CI) = 2.0–4.3, digital cellular phones OR = 1.5, 95 % CI = 1.1–2.1 and cordless phones OR = 1.5, 95 % CI = 1.04–2.0. The corresponding results were for astrocytoma grade III-IV OR = 1.7, 95 % CI = 1.3–2.3; OR = 1.5, 95 % CI = 1.2–1.9 and OR = 1.5, 95 % CI = 1.1–1.9, respectively. The ORs increased with latency period with highest estimates using > 10 years time period from first use of these phone types. Lower ORs were calculated for astrocytoma grade I-II. No association was found with salivary gland tumours, NHL or testicular cancer although an association with NHL of T-cell type could not be ruled out.

**Conclusion:**

We found for all studied phone types an increased risk for brain tumours, mainly acoustic neuroma and malignant brain tumours. OR increased with latency period, especially for astrocytoma grade III-IV. No consistent pattern of an increased risk was found for salivary gland tumours, NHL, or testicular cancer.

## Background

During the most recent decades there has been a rapid development of the use of wireless telephone communication. The Nordic countries in Europe were among the first in the world to introduce this new technology.

The analogue (NMT; Nordic Mobile Telephone System) phones operating at 450 MegaHertz (MHz) were introduced in Sweden in 1981. In the beginning they were usually used in a car with fixed external antenna. Portable NMT 450 phones were introduced in 1984. Analogue phones using 900 MHz (NMT 900) were used in Sweden between 1986 and 2000. The digital system (GSM; Global System for Mobile Communication) started in 1991 and has during recent years dramatically increased to be the most common phone type. This system uses dual band, 900 and 1 800 MHz, for communication. From 2003 the third generation of mobile phones, 3G or UMTS (Universal Mobile Telecommunication System) has started in Sweden operating at 1 900 MHz.

Desktop cordless phones also use wireless technology. First the analogue system in the 800–900 MHz RF was used when these phones were available in Sweden in 1988. Digital cordless telephones (DECT) that operate at 1900 MHz are used since 1991.

Use of mobile and desktop cellular telephones results in exposure to microwaves. Exposure is characterized through the specific absorption rate (SAR) expressed as watt/kg. The anatomical area with the highest exposure is the ipsilateral (same) side of the brain that is used during the call. If a hands-free device is used and the cellular telephone is placed at another part of the body that anatomical area receives the highest radio frequency (RF) exposure. The cellular telephone communicates with a base station usually located at some distance, the antenna of which typically is on the top of a building or on a mast. Several workplaces use only cellular or cordless phones instead of the landline phones and this leads to both active and passive exposure to microwaves during the working day for the employees. Very few workplaces offer hands free devices to the employees although the Nordic radiation protection authorities as well as the Swedish work environmental board recommend this.

The introduction of wireless communication has been technically driven without proper laboratory testing or epidemiological studies of potential health effects. Among the first to express concern of adverse health effects due to exposure to microwaves from cellular phones was the layman [1]. At that time the technology was rather new and the use of cell phones was not so widespread. The large expansion has occurred since late 1990's. Now 200 million persons are users in USA and in Sweden almost everyone has a cellular phone. Thus, even a health problem of little magnitude would give serious consequences in the society due to the large number of exposed persons.

Since the second part of the 1990's we have performed six case-control studies on this topic encompassing use of both cellular and cordless phones as well as other exposures. This is an overview of the findings in these studies. Three of our studies concerned brain tumours. The first one was rather small [2,3]. This was followed by two larger case-control studies on brain tumours [4-7]. Here we present results from the pooled analysis of these two studies [8,9]. Because of the anatomical localization of salivary glands, especially the parotid, in an area with high exposure to microwaves during calls, we performed also a case-control study on salivary gland tumours [10].

During the same time we studied risk factors for non-Hodgkin lymphoma (NHL), mainly to elucidate pesticide exposure as discussed elsewhere [11]. In that study we also included similar questions on the use of cellular and cordless phones [12] as in our at the same time on-going studies on brain tumours. NHL might be of interest in this context due to potential effects on the immune system from microwaves [13,14], since immune modulation is a risk factor in lymphomagenesis [11]. Also certain cutaneous forms of NHL might be of concern due to skin absorption of microwaves during phone calls.

Finally we have also studied testicular cancer, the main topic being chemical exposures, e.g., polyvinyl chloride [15]. The results regarding use of cellular and cordless telephones have not been published so far. It might be argued that the testes are at some distance from the cellular or cordless phone during calls. However, there has been some concern in the population that keeping the phone in a pocket might be a risk factor for testicular cancer. A recent study found a moderate correlation between mobile phone use and semen quality [16].

In the following a short description of the studies is given, further details are displayed in the various publications. In principle the same epidemiological methods were used in all studies.

## Materials and methods

All studies were performed in Sweden covering various health service regions and at somewhat different time periods for recruitment of cases and controls, see Table 1. The studies on NHL, brain and salivary gland tumours included both sexes. The Cancer Registries in Sweden were used to ascertain the cases. The treating physicians were contacted to get permission to include the cases in the studies. Deceased cases were excluded from the studies, mainly patients with malignant brain tumours having a bad prognosis. The controls were population based drawn from the Swedish Population Registry covering the whole country. They were matched on sex, age and geographical area, i.e., the same geographical area as for the cases in the different investigations. Each study person was given a unique ID number that did not reveal whether the person was a case or a control.

**Table 1:** Description of studies by Hardell et al on use of cellular and cordless telephones and the risk for tumour diseases.

**Study**	**Geographical area**	**Years**	**Included persons**	**Response rate**
CNS [2,3]	Uppsala/Örebro Stockholm	1994–1996 1995–1996	233 cases* 466 controls	209 (90%) cases 425 (91%) controls
CNS [4,5]	Uppsala/Örebro, Stockholm, Linköping, Göteborg	Jan 1, 1997 – June 30, 2000	1617 cases 1617 controls	1429 (88%) cases 1470 (91 %) controls
CNS, benign [6]	Uppsala/Örebro, Linköping	July 1, 2000 – Dec 31, 2003	462 cases** 820 controls	413 (89 %) cases 692 (84 %) controls
CNS, malignant [7]	Uppsala/Örebro, Linköping	July 1, 2000 – December 31, 2003	359 cases** 820 controls	317 (88 %) cases 692 (84 %) controls
Salivary gland tumours [10]	Stockholm, Linköping Uppsala/Örebro Umeå, Göteborg, Lund	Jan 1, 1994 – Dec 31, 1999 Jan 1, 1994 – June 30, 2000 Jan 1, 1994 – June 30, 1999	293 cases 1172 controls	267 (91%) cases 1053 (90%) controls
Non-Hodgkin lymphoma [11]	Umeå, Örebro, Linköping, Lund	Dec 1, 1999 – April 30, 2002	995 cases 1108 controls	910 (91%) cases 1016 (92%) controls
Testicular cancer Hardell et al to be published	Whole Sweden	1993 – 1997	981 cases 981 controls	889 (91%) cases 870 (89%) controls

* One case had two benign brain tumours.

** One case had both a malignant and a benign brain tumour

### Assessment of exposure

All investigations were approved by the responsible ethical committees and were performed according to the ethical standards laid down by the Helsinki Declaration. All included persons had the possibility to refuse participation. Exposures were assessed by mailed questionnaires and the answers were supplemented over the phone by a trained interviewer using a structured protocol. The interviews as well as coding of the answers for statistical analyses were made blinded as to case or control status. Details have been further explored in the various publications. It should be noted that use of cordless phones was not assessed in our first brain tumour study [2,3].

### Statistical analysis

Odds ratios (OR) and 95 % confidence intervals (CI) (SAS Institute, Cary, NC) were calculated using conditional logistic regression analysis in the first study on cellular telephones and brain tumour risk [2,3]. In the following studies unconditional logistic regression analysis was performed (Stata/SE 8.2 for Windows; StataCorp, College Station, TX). The unexposed category consisted of subjects that had not used cellular or cordless phones. The exposed cases and controls were divided according to phone type, analogue, digital and cordless. Note that the analyses were made for those who anytime (disregarding 1 year latency period) had used an analogue or digital cellular phone or a cordless phone. However, it is common that many users have been using all three systems, see further the discussion section. Exposure the year before diagnosis was thus disregarded in the assessment of exposure. Thereby the same year for diagnosis of the case was used for the corresponding control as cut-off for exposure. Thus exposure the year before the diagnosis of the case was also disregarded for the control. Adjustment was made for sex, age, socio-economic index (SEI)-code and year for diagnosis in the analysis of the two next brain tumour case-control studies [8,9]. Adjustment for year of diagnosis was made in order to avoid bias in exposure since all controls both to malignant and benign brain tumour cases were used in the analyses. We used age as a continuous variable in the analysis.

In the study on NHL adjustment was made for age, sex and year of diagnosis (cases) or enrolment (controls). The results in the testicular cancer study were adjusted for age and cryptorchidism.

Latency or tumour induction period was in this presentation analysed using three time periods, > 1 year, > 5 years and > 10 years since first use of a cellular or cordless telephone until diagnosis. In the dose-response calculations median number of cumulative lifetime use in hours among controls was used as cut-off. Regarding brain tumours calculation of trend was made dividing cumulative use among the controls in tertiles.

## Results

The response rates in the different studies were high, see Table 1. In the following results for the different diseases are discussed.

### Brain tumours

In our first study no increased risk was found overall, see Table 2[2]. However, ipsilateral exposure adjusted for other risk factors, laboratory work and medical diagnostic X-ray investigations of the head and neck region, yielded OR 2.6, 95 % CI 1.02–6.7 for brain tumours (benign and malignant together) in the temporal, occipital or temporoparietal lobes, i.e. most exposed areas [3]. Only 16 cases had used an analogue cellular phone for > 10 years. Digital phones had been used by 4 cases with a latency period > 5 years and no case for > 10 years. Thus, this study was limited by low numbers of exposed cases and short latency periods and no firm conclusions could be drawn.

**Table 2:** Use of cellular and cordless phones and odds ratio (OR) and 95 % confidence intervals (CI) for different tumour types.

	**> 1 year latency**			**> 5 years latency**			**> 10 years latency**		
Study, period	Analogue OR CI	Digital OR CI	Cordless OR CI	Analogue OR CI	Digital OR CI	Cordless OR CI	Analogue OR CI	Digital OR CI	Cordless OR CI

CNS 1994–1996 [2,3]									
-All	0.9 0.6–1.4	1.0 0.6–1.5	NA	0.8 0.5–1.4	0.3 0.03–2.1	NA	1.2 0.6–2.6	-*	NA
CNS 1997–2003 [8,9]									
-All	1.5 1.3–1.9	1.2 1.03–1.4	1.3 1.1–1.5	1.7 1.3–2.1	1.6 1.2–2.0	1.6 1.3–1.9	2.2 1.6–3.0	2.8 1.5–5.3	1.9 1.2–2.9
-Benign, all	1.6 1.3–2.0	1.2 0.96–1.4	1.2 1.01–1.4	1.7 1.3–2.3	1.3 0.99–1.8	1.5 1.2–1.9	1.9 1.3–2.8	2.2 0.99–4.9	1.6 0.9–2.7
-Meningoma	1.3 0.99–1.7	1.1 0.9–1.3	1.1 0.9–1.4	1.3 0.98–1.8	1.2 0.9–1.7	1.5 1.1–1.9	1.6 1.04–2.6	1.8 0.7–4.6	1.8 1.01–3.2
-Acoustic neuroma	2.9 2.0–4.3	1.5 1.1–2.1	1.5 1.04–2.0	3.2 2.0–4.9	1.6 0.9–2.8	1.6 1.02–2.6	3.2 1.7–6.1	0.8 0.1–6.6	1.3 0.4–3.8
-Malignant, all	1.5 1.1–1.9	1.3 1.1–1.6	1.3 1.1–1.6	1.6 1.2–2.1	1.8 1.3–2.4	1.6 1.2–2.1	2.4 1.7–3.5	3.4 1.6–7.3	2.1 1.2–3.5
-Astrocytoma, grade I-II	1.2 0.6–2.2	1.4 0.9–2.3	1.4 0.9–2.3	1.1 0.5–2.3	1.5 0.7–3.3	1.9 1.03–3.5	1.5 0.6–4.1	1.3 0.1–13	1.8 0.6–5.9
-Astrocytoma, grade III-IV	1.7 1.3–2.3	1.5 1.2–1.9	1.5 1.1–1.9	1.9 1.4–2.7	2.5 1.7–3.5	2.1 1.5–2.9	3.0 2.0–4.6	5.2 2.2–12	2.7 1.5–5.0
Salivary gland tumours 1994–1999 [10]									
-All	0.9 0.6–1.4	1.0 0.7–1.5	1.0 0.7–1.4	0.8 0.4–1.4	1.2 0.5–2.8	1.1 0.6–2.0	0.7 0.3–1.7	-*	-*
Non-Hodgkin lymphoma 1999–2002 [11]									
-B-cell	0.9 0.7–1.3	1.0 0.8–1.3	1.0 0.8–1.3	1.0 0.7–1.4	0.9 0.7–1.3	1.0 0.7–1.3	1.0 0.7–1.4	1.1 0.4–3.3	1.1 0.6–1.8
-T-cell	1.6 0.6–3.8	1.4 0.7–2.9	1.4 0.6–2.9	1.5 0.6–3.7	1.9 0.8–4.8	2.5 1.1–5.6	1.5 0.5–4.3	3.0 0.3–34	3.2 1.05–9.5
Testicular cancer Hardell et al, to be published 1993–1997									
-All	1.0 0.8–1.3	1.2 0.9–1.6	1.1 0.8–1.4	1.2 0.9–1.8	3.4 0.9–13	1.1 0.7–1.6	1.6 0.7–3.8	-*	-*
-Seminoma	1.2 0.9–1.6	1.3 0.9–1.8	1.1 0.8–1.5	1.5 0.98–2.2	4.1 0.97–17	1.2 0.7–1.9	2.1 0.8–5.0	-*	-*
-Non-seminoma	0.7 0.5–1.1	0.9 0.6–1.4	1.0 0.7–1.4	0.9 0.5–1.6	2.3 0.5–12	0.9 0.5–1.6	0.3 0.04–2.7	-*	-*

Results are given for different latency periods.

*No exposed cases.

NA = not assessed

The following two case-control studies on brain tumours were larger and encompassed answers from 1 254 (88 %) of cases with benign brain tumour, 905 (90 %) with malignant brain tumour and 2 162 (89 %) controls. Here results are given from the pooled analysis of these two case control studies [8,9]. Detail from the separate studies can be found elsewhere [4-7].

Regarding meningioma the risk increased with latency period. With latency > 10 years analogue phones yielded OR 1.6, 95 % CI 1.04–2.6, digital phones OR 1.8, 95 % CI 0.7–4.6 and cordless phones OR 1.8, 95 % CI 1.01–3.2. However, in the multivariate analysis adjusted for the different phone types lower ORs were found and no was statistically significant [8].

All phone types increased the risk for acoustic neuroma. Regarding analogue phones OR increased with latency period and was highest in the category with latency period > 15 years yielding OR = 3.5, 95 % CI = 1.4–10 [8]. Increased risk was also found for digital cellular telephones and cordless phones. However, in the multivariate analysis only analogue phones were significant risk factors with OR 2.2, 95 % CI 1.3–3.8 using > 10 year latency period [8].

In Table 3 results are displayed for use in hours divided in tertiles based on use among controls. For the whole group of benign tumours a significant trend was found for total use in any combination of the different phones. Regarding meningioma no significant trend was found whereas for acoustic neuroma and the group of other benign tumours total use yielded a significant trend.

**Table 3:** Odds ratio (OR) and 95 % confidence interval (CI) for cumulative lifetime use in hours of analogue and digital cellular telephones, cordless telephones and any combination of the three phone types for benign brain tumours [8].

	**First tertile (h)**			**Second tertile (h)**			**Third tertile (h)**		
	Ca/Co	OR	95 % CI	Ca/Co	OR	95 % CI	Ca/Co	OR	95 % CI
	
**Benign**									
Analogue	77/109	1.5	1.1–2.1	51/89	1.4	0.97–2.1	71/99	1.9	1.3–2.7
Digital	175/283	1.2	0.9–1.5	141/246	1.2	0.9–1.5	121/247	1.1	0.9–1.5
Cordless	146/264	1.1	0.9–1.4	108/204	1.1	0.8–1.4	169/233	1.5	1.2–1.9
Total, any combination	238/405	1.1	0.9–1.4	187/377	1.0	0.8–1.2	252/390	1.4	1.1–1.7
**Meningioma**									
Analogue	47/109	1.3	0.9–1.9	32/89	1.3	0.8–2.0	34/99	1.3	0.9–2.1
Digital	116/283	1.0	0.8–1.3	106/246	1.2	0.9–1.6	73/247	1.0	0.7–1.4
Cordless	106/264	1.1	0.8–1.4	70/204	0.9	0.7–1.3	118/233	1.4	1.1–1.9
Total, any combination	163/405	1.0	0.8–1.3	136/377	1.0	0.8–1.2	162/390	1.2	0.96–1.6
**Acoustic neuroma**									
Analogue	20/109	2.3	1.3–4.0	18/89	2.7	1.5–4.9	30/99	4.1	2.4–7.0
Digital	42/283	1.7	1.1–2.5	26/246	1.2	0.7–1.9	37/247	1.7	1.03–2.7
Cordless	27/264	1.1	0.7–1.8	29/204	1.5	0.95–2.5	40/233	1.8	1.2–2.8
Total, any combination	46/405	1.3	0.9–1.9	40/377	1.2	0.8–1.8	69/390	2.0	1.4–3.0
**Other benign**									
Analogue	10/109	2.5	1.2–5.5	2/89	0.5	0.1–2.1	7/99	1.7	0.7–4.2
Digital	17/283	2.1	1.1–3.9	10/246	1.2	0.5–2.6	11/247	1.0	0.4–2.1
Cordless	13/264	1.4	0.7–2.8	9/204	1.4	0.6–3.2	12/233	1.5	0.7–3.1
Total, any combination	29/405	2.1	1.2–3.5	11/377	0.8	0.4–1.7	22/390	1.5	0.8–2.7

Number of exposed cases (Ca) and controls (Co) are given. Unconditional logistic regression analysis adjusted for age, sex, socio-economic index and year of diagnosis was used. Tertiles were based on use among controls.

*Trend, benign: Analogue – p = 0.42, digital – p = 0.97, cordless – p = 0.06, total – p = 0.02*.

*Trend, meningioma: Analogue – p = 0.99, digital – p = 0.40, cordless – p = 0.07, total – p = 0.18*.

*Trend, acoustic neuroma: Analogue – p = 0.17, digital – p = 0.31, cordless – p = 0.18, total – p = 0.02*.

*Trend, other benign: Analogue – p = 0.11, digital – p = 0.16, cordless – p = 0.98, total – p = 0.047*.

Analogue: First tertile – 1–43 h, second tertile – >43–165 h, third tertile – >165 h

Digital: First tertile – 1–30 h, second tertile – >30–149 h, third tertile – >149 h

Cordless: First tertile – 1–122 h, second tertile – >122–365 h, third tertile – >365 h

Total, any combination: First tertile – 1–91 h, second tertile – >91–410 h, third tertile – >410 h

For astrocytoma grade I-II there was no clear trend of increasing OR with increasing latency period, see Table 2. Cordless phones yielded OR 1.9 of borderline significance with latency > 5 years but OR did not increase further with latency > 10 years and was not statistically significant in that group.

On the contrary, for astrocytoma grade III-IV OR increased with latency period and was highest using > 10 year latency for all phone types. In that latency group multivariate analysis yielded for analogue phones OR 2.0, 95 % CI 1.4–2.9, digital phones OR 2.4, 95 % CI 1.1–4.9 and cordless phones OR 1.3, 95 % CI 0.8–2.3 [9].

Trend test gave for all malignant tumours together and astrocytoma grade III-IV a significant result for cordless phones and total use in any combination of the different phone types, see Table 4. No significant trend was obtained for astrocytoma grade I-II or other types of malignant tumours.

**Table 4:** Odds ratio (OR) and 95 % confidence interval (CI) for cumulative lifetime use in hours of analogue and digital cellular telephones, cordless telephones and any combination of the three phone types for malignant brain tumours [9].

	**First tertile (h)**			**Second tertile (h)**			**Third tertile (h)**		
	Ca/Co	OR	95 % CI	Ca/Co	OR	95 % CI	Ca/Co	OR	95 % CI
	
**Malignant**									
Analogue	57/109	1.4	0.97–2.0	41/89	1.1	0.7–1.7	80/99	1.9	1.3–2.7
Digital	133/283	1.3	1.03–1.7	108/246	1.1	0.9–1.5	161/247	1.5	1.1–1.9
Cordless	107/264	1.1	0.8–1.4	94/204	1.2	0.9–1.6	149/233	1.7	1.3–2.3
Total, any combination	170/405	1.2	0.9–1.5	169/377	1.2	0.9–1.5	244/390	1.5	1.2–1.9
**Astrocytoma, grade I-II**									
Analogue	5/109	0.9	0.3–2.4	7/89	1.4	0.6–3.5	7/99	1.3	0.5–3.3
Digital	20/283	1.6	0.9–2.9	12/246	1.0	0.5–2.0	24/247	1.6	0.8–2.9
Cordless	15/264	1.2	0.6–2.2	13/204	1.1	0.5–2.2	28/233	1.9	1.1–3.5
Total, any combination	25/405	1.4	0.8–2.4	22/377	1.1	0.6–2.0	41/390	1.7	1.04–2.9
**Astrocytoma, grade III-IV**									
Analogue	34/109	1.5	0.99–2.4	27/89	1.3	0.8–2.2	54/99	2.3	1.5–3.5
Digital	74/283	1.4	1.01–1.9	71/246	1.4	1.02–2.0	99/247	1.8	1.3–2.5
Cordless	65/264	1.2	0.8–1.6	50/204	1.3	0.9–1.9	90/233	2.1	1.5–2.9
Total, any combination	91/405	1.1	0.8–1.5	104/377	1.4	1.03–1.8	146/390	1.8	1.3–2.4
**Other malignant**									
Analogue	18/109	1.5	0.8–2.6	7/89	0.7	0.3–1.7	19/99	1.6	0.9–3.0
Digital	39/283	1.4	0.9–2.1	25/246	0.9	0.5–1.4	38/247	1.1	0.7–1.8
Cordless	27/264	1.0	0.6–1.6	31/204	1.3	0.8–2.1	31/233	1.2	0.8–1.9
Total, any combination	54/405	1.4	0.9–2.0	43/377	1.1	0.7–1.6	57/390	1.3	0.9–1.9

Number of exposed cases (Ca) and controls (Co) are given. Unconditional logistic regression analysis adjusted for age, sex, socio-economic index and year of diagnosis was used. Tertiles were based on use among controls.

*Trend, malignant: Analogue – p = 0.11, digital – p = 0.21, cordless – p = 0.01, total – p = 0.04*.

*Trend, astrocytoma, grade I-II: Analogue – p = 0.72, digital – p = 0.38, cordless – p = 0.16, total – p = 0.30*.

*Trend, astrocytoma, grade III-IV: Analogue – p = 0.10, digital – p = 0.26, cordless – p = 0.01, total – p = 0.01*.

*Trend, other malignant: Analogue – p = 0.21, digital – p = 0.23, cordless – p = 0.64, total – p = 0.50*.

Analogue: First tertile – 1–43 h, second tertile – >43–165 h, third tertile – >165 h

Digital: First tertile – 1–30 h, second tertile – >30–149 h, third tertile – >149 h

Cordless: First tertile – 1–122 h, second tertile – >122–365 h, third tertile – >365 h

Total, any combination: First tertile – 1–91 h, second tertile – >91–410 h, third tertile – >410 h

Many people in the study had been using all three types of phones: NMT, GSM and cordless. The most obvious combination of the use of different phones is to add the total time on each phone without setting different weight to each of them. However, the different phone types have different output power. The NMT phone is operating with a maximum power of 1 W and very seldom down regulates this; the GSM 900 phone is operating with a maximum of 0.25 W but can down regulate the power to a few mW depending on the distance to the base station, and a typical value would be 0.1 W; the cordless phones operate at 10 mW. One selection of weighting factors according to mean output power of the phones could then be NMT = 1, GSM = 0.1, and cordless = 0.01 [17,18]. These factors have been used in Table 5 where the time spent on each of the phone types has been multiplied with these factors before adding them into one score using data in our second brain tumour study [4,5]. The results differ depending on how the combination is done, but not so much. The main trend with an increased risk with increased hour of use is also seen in these calculations, obvious in the > 10 year latency group [18].

**Table 5:** Odds ratio (OR) and 95 % confidence interval (CI) for brain tumours [4,5].

**> 1 year latency**				**> 5 years latency**				**> 10 years latency**			
Score	Ca/Co	OR	95 % CI	Score	Ca/Co	OR	95 % CI	Score	Ca/Co	OR	95 % CI

Total	716/713	1.1	0.9 – 1.3	Total	321/272	1.3	1.1 – 1.6	Total	69/51	1.6	1.1 – 2.3
≤ 11.0	350/357	1.0	0.9 – 1.3	≤ 46.8	167/136	1.3	1.04 – 1.7	≤ 166	29/26	1.2	0.7 – 2.1
> 11.0	366/356	1.1	0.9 – 1.3	> 46.8	154/136	1.3	0.98 – 1.7	> 166	40/25	1.9	1.1 – 3.3

Score by multiplying weighting factors, analogue= 1, digital = 0.1, cordless telephone = 0.01, with cumulative use in hours for the different phone types and adding all three categories was used. Unconditional logistic regression analysis adjusted for age, gender and socioeconomic index was used. Unexposed groups were used for comparison. Median score among the controls for each latency period used as cut-off. Number of cases (Ca) and controls (Co) is given.

In Table 6 results are presented for ipsilateral exposure using > 1 year latency period. Highest ORs were found for acoustic neuroma and astrocytoma grade III-IV for both cellular and cordless desktop phones. Digital mobile phones yielded for meningioma and astrocytoma grade I-II increased OR of borderline significance. Also cordless phones gave for astrocytoma grade I-II increased OR of borderline significance.

**Table 6:** Odds ratio (OR) and 95 % confidence interval (CI) for ipsilateral use of mobile (analogue, digital) or cordless phones.

	**Ipsilateral**		
Study, period	Analogue OR CI	Digital OR CI	Cordless OR CI

CNS 1997–2003 [8,9]			
-All	2.0 1.5–2.6	1.6 1.3–2.0	1.5 1.2–1.9
-Benign, all	1.9 1.3–2.6	1.5 1.1–1.9	1.4 1.1–1.8
-Meningioma	1.3 0.9–2.0	1.4 1.01–1.8	1.3 0.9–1.7
-Acoustic neuroma	3.0 1.9–5.0	1.7 1.1–2.6	1.7 1.1–2.6
-Malignant, all	2.1 1.5–2.9	1.8 1.4–2.4	1.7 1.3–2.2
-Astrocytoma, grade I-II	1.8 0.8–4.1	1.9 1.02–3.5	1.9 1.05–3.5
-Astrocytoma, grade III-IV	2.4 1.6–3.6	2.3 1.7–3.1	2.0 1.5–2.8
Salivary gland tumours 1994–1999 [10]			
-All	1.4 0.8–2.6	1.2 0.7–2.0	1.0 0.6–1.7

Results are presented for brain tumours [8,9] and salivary gland tumours [10] using > 1 year latency period.

### Salivary gland tumours

No association between use of cellular or cordless phones and salivary gland tumours was found [10]. The results were limited due to few cases with long-term use of the phone types. Only 6 cases had used an analogue phone > 10 years and no one had used a digital or cordless phone using that latency period. Thus, further studies would be necessary to make definitive conclusions regarding an association. No significantly increased OR was found for ipsilateral exposure, Table 6.

### Non-Hodgkin lymphoma (NHL)

No association was found with B-cell NHL [12]. Regarding T-cell NHL OR increased with latency period for digital and cordless phones. Latency period > 5 years for use of analogue cellular phones yielded OR = 1.5, 95 % CI = 0.6–3.7, digital OR = 1.9, 95 % CI = 0.8–4.8, and cordless phones OR = 2.5, CI = 1.1–5.6. The corresponding results for extranodal T-cell lymphoma were for analogue phones OR = 3.4, 95 % CI = 0.8–15.0, digital OR = 6.1, 95 % CI = 1.3–29.7 and cordless phones OR = 5.5, 95 % CI = 1.3–23.9.

### Testicular cancer

Results from this study have so far only been published for exposure to polyvinyl chloride, which was the main issue of the study [15]. Questions on use of cellular and cordless phones were also included in the questionnaire in the similar way as in the other studies above. However, this time we also included questions on where the cellular phone usually was kept between calls. We asked if the phone was on stand-by during that time. The results were based on answers from 542 (92 %) cases with seminoma, 346 (89 %) with non-seminoma and 870 (89 %) controls. Regarding seminoma use of analogue cellular phones gave OR = 1.2, 95 % CI = 0.9–1.6, digital phones OR = 1.3, CI = 0.9–1.8, and cordless phones OR = 1.1, CI = 0.8–1.5. The corresponding results for non-seminoma were OR = 0.7, CI = 0.5–1.1, OR = 0.9, CI = 0.6–1.4, and OR = 1.0, CI = 0.7–1.4, respectively. A somewhat increased OR was found for seminoma and use of analogue phones in the group with > 5 year latency period yielding OR = 1.5, 95 % CI = 0.98–2.2 and for digital phones with OR = 4.1, 95 % CI = 0.97–17, and cordless phones OR = 1.2, 95 % CI = 0.7–1.9. Regarding non-seminoma digital phones yielded in the same category OR = 2.3, 95 % CI = 0.5–12 whereas OR for analogue cellular phones and cordless phones was close to unity. No association was found with place of keeping the mobile phone during stand-by, such as trousers pocket. Cryptorchidism was a risk factor for both seminoma and non-seminoma, but no interaction with cellular or cordless phones was found.

## Discussion

The same study methods were used in all these case-control studies performed by our research group. The results varied for different tumour types and would thus not be expected to be caused by observational or recall bias since such bias should have existed for all tumour types. Moreover the results seem to be of biological relevance regarding tumour type, tumour localisation, latency period and dose-response effect.

Cases were ascertained from the Swedish Cancer Registry that has a good coverage of all new cases. Controls were enrolled from the Swedish Population Registry that covers the whole population. All subjects in Sweden have a unique id-number. Thus, no selection bias was introduced in the enrolment of cases and controls in the various studies. The population registry also makes it possible to find the address of all included subjects so no case or control was excluded due to lack of address for mailing of the questionnaire. It should however be noted that only living cases were included in the studies. Of brain tumours glioblastoma multiforme has a bad prognosis. This may have shifted the distribution of histopathological types of cases to slightly better prognosis. The influence on the results, if any, is currently unknown.

Regarding brain tumours assessment of exposure was made about two months after histopathological diagnosis. One advantage was that the cases were informed about their diagnoses and that the cases could answer to the questionnaires and phone interviews at home in a more relaxed setting than in a hospital. When supplementing the data in the questionnaires over the phone it was not revealed if it was a case or a control. The coding of the data for statistical analysis was made without knowing the identity of the subject. Thus, observational bias was avoided in the studies.

In the brain tumour studies we found the highest OR for acoustic neuroma. This tumour might be a "signal" tumour type for increased brain tumour risk from microwave exposure, since it is located in an anatomical area with high exposure during calls with cellular or cordless phones. In fact, an increasing incidence of acoustic neuroma has been noted in Sweden [19]. For both analogue cellular telephones and cordless desktop phones the risk was highest in the third tertile of use in hours. However, no such trend was seen for digital phones. For all phones combined we found a significant trend of OR with increasing time for use, *p *= 0.02.

Regarding meningioma no significant trend was found. Cordless phones produced highest OR in the third tertile of borderline significance. For use of any phone no significantly increased risk was found. OR was highest for other types of benign tumours in the first tertile for use of analogue or digital phones. For cordless phones the OR was similar in all three categories of use. Thus, the results for other types of benign brain tumours indicate that there is no association and that longer follow-up time is needed for evaluation of long-term effects.

For astrocytoma grade I-II highest OR was calculated in the third tertile of use in hours, see Table 4. ORs were statistically significantly increased for cordless phones and total use in any combination. The trend tests of these categories of exposure were not significant, however.

Regarding astrocytoma grade III-IV significantly increased risks were found in the highest exposure category, see Table 4. As presented elsewhere [9] both analogue and digital cellular telephones were statistically significant risk factors in the multivariate analysis. However, in the trend test of cumulative use the result was statistically significant only for cordless telephones and total use of all phone types together, see Table 4.

Adaptive power control (APC) gives a difference in power output from mobile phones between urban and rural areas due to regulations of the emissions by the distance to the base stations. The place of residence for the cases and controls in our second brain tumour study [4,5] was divided in groups based on population density using Statistics Sweden [20]. A clear effect was seen for digital phone users with highest risk in rural areas, OR = 3.2, 95 % CI = 1.2–8.4, compared with in urban areas OR = 0.9, 95 % CI = 0.6–1.4, using > 5 year latency period. The power output is highest in rural areas so the results indicate a dose-response effect. For analogue phones no such pattern was found that might be explained by the fact that APC has not previously been used for analogue phones.

The same study method as in the brain tumour studies was used for salivary gland tumours [10]. We did not find an association between use of cellular or cordless telephones and salivary gland tumours in this study. There was no effect with increasing tumour induction period or number of hours of use of the different phones. However, only 6 cases had used a phone for more than 10 years, and all of these subjects had used the analogue type. Thus, this study cannot exclude an increased risk among subjects with heavy use for a long time period. The power of the study was to detect an OR ≥ 1.4 (α = 0.05, β = 0.20). This case-control study was performed during the same time period as our brain tumour studies. These results strongly argue against observational and recall bias as the explanation for our results in the brain tumour studies. A recent study did not find an association between mobile phone use and parotid gland tumour regardless of duration of use in hours or years since first use [21].

The results in our case-control study on NHL are of potential interest [11]. We found no association with B-cell lymphoma whereas the findings for T-cell NHL may be of importance. Analysing the cutaneous and leukaemia types of T-cell NHL increased the risk further. T-cell NHL is uncommon and represented 5.8 % in our study. T-cell lymphomas are derived from mature or post-thymic circulating T-cells. Exposure to microwaves may occur in the circulating blood during a phone call. Our results were based on low numbers and must be interpreted with caution. There is no obvious biological mechanism that explains the results and further studies are therefore necessary.

The main result of the study on testicular cancer was no association with use of cellular or cordless telephones [Hardell et al, to be published]. For seminoma significantly increased OR was calculated in lowest exposure category with > 1 year latency period for all studied phone types. However, there was no dose-response effect and no significant trend for increasing OR with increasing latency period. As one would expect cryptorchidism was associated with increased risk for both seminoma and non-seminoma but did not interact with use of cellular or cordless phones. The localization of the mobile phone during stand-by time was also analyzed. However, no association was found with testicular cancer. Keeping the phone in a pocket close to the testis did not increase the risk and there was no association with laterality of the phone and cancer.

In studies of tumour risk and mobile phone use exposure assessment becomes an even greater problem than for the acute effects since for this type of disease it is the exposure 5–10 years or more ago that is of interest. Most users of mobile phones have not been using just one single telephone. It is even more likely that if they have been using a mobile phone for more than a few years, they will also have changed their phone a few times. Many users will also have used different phone systems such as analogue and digital, and probably many of them have also been using a cordless phone at home or at work. The problem we are facing is then how to integrate the various SAR distributions from the different devices and add up the different times on these phones to one exposure measure? At the moment it is not clear how to combine the use of different phones with different power output, different systems, different frequencies, and different anatomical SAR distribution, into one exposure and dose measure. The difficulties lay in the fact that we do not know the interacting mechanism(s) between the electromagnetic fields emitted from the phone and the biological organism.

We used a weighting method as described above to combine exposure measurement from different phone types; NMT = 1, GSM = 0,1 and cordless phones = 0.01. This method was applied for data in our second brain tumour study [4,5] and has been discussed elsewhere [17,18]. The results did not differ much from using no weighting factor. This could be due to the large weight put to the NMT phone due to their high output power. On theoretical ground, using the sum of the use in hours of the different phone types is obviously not an appropriate method when combining exposure to these radio frequency (RF) fields. Using a weighting factor might be appropriate until a proper dosimetry is available.

In future epidemiological studies on brain tumours an important consideration ought to be which time scale to use, and this must be based on hypotheses about induction and progression of the endpoint variables being studied. One needs to set up a clear hypothesis about how the absorption to RF from mobile phones could influence the endpoint variable in terms of anatomical localization of the absorption, the duration of the exposure and the induction and progression of the endpoint variable before choosing an appropriate dosimetric quantity.

One of the questions we need to address is for instance how time comes into the connection between exposure and dose, and here we need to distinguish between different aspects of time: very short times – order of minutes, daily averages, and total time in the actual occupation – number of years with exposure. Another question that is urgent to address is the potential for greater biological effects from RF fields in young age groups. We have found some indication for that with higher risk for brain tumours in persons with first use of cellular or cordless phones before the age of 20 years compared with older ages [8,9,22].

## Conclusion

We have here presented results from our studies on this topic. The intention was not to cover the whole area, such presentations can be found in other publications [23-25]. In our series of studies on tumour risk associated with use of cellular or cordless telephones the consistent finding for all studied phone types was an increased risk for brain tumours, mainly acoustic neuroma and malignant brain tumours. Using a latency period of > 10 years ORs increased especially for astrocytoma grade III-IV. No consistent pattern of an increased risk was found for salivary gland tumours, NHL or testicular cancer.

## Competing interests

The author(s) declare that they have no competing interests.

## Authors' contributions

**LH **was the principal investigator responsible for the design, conduct and interpretation of the studies.

**KHM **participated in all aspects of the studies, especially with his technical knowledge.

**MC **participated as a statistician in all parts of the studies.

**FS **participated in the compilation and interpretation of the data for this publication.
